# Pseudogene *DUXAP8* Promotes Cell Proliferation and Migration of Hepatocellular Carcinoma by Sponging MiR-490-5p to Induce BUB1 Expression

**DOI:** 10.3389/fgene.2020.00666

**Published:** 2020-07-22

**Authors:** Haiping Zhang, Kaiqiu Chu, Chunxi Zheng, Lisheng Ren, Runhua Tian

**Affiliations:** ^1^Department of Clinical Laboratory, The Affiliated Hospital of Qingdao University, Qingdao, China; ^2^Department of Clinical Laboratory, Qingdao Women and Children’s Hospital, Qingdao, China

**Keywords:** Hepatocellular Carcinoma, long non-coding RNA, bioinformatics, regulatory mechanism, target

## Abstract

Hepatocellular Carcinoma (HCC) currently remains one of the most lethal malignancies worldwide. Recently, long non-coding RNAs (lncRNAs) had been demonstrated to play a crucial role in the progression of multiple human cancers, including HCC. In this study, we found that lncRNA DUXAP8 was upregulated in tumor samples and served as an oncogene in HCC. Bioinformatics analysis showed that DUXAP8 was significantly associated with the regulation of centrosome organization, homologous recombination, meiotic cell cycle process, sister chromatid segregation, nuclear chromosome segregation, and RNA export from the nucleus. The knockdown of DUXAP8 significantly suppresses cell proliferation and the cell cycle but induces cell apoptosis in HCC. Mechanically, the present study showed that DUXAP8 serves as a sponge of MiR-490-5p to promote the expression of BUB1 in HCC. Although the underlying regulatory mechanisms of DUXAP8 in HCC require further investigation, this study, for the first time, showed that DUXAP8 can serve as a new therapeutic target for HCC.

## Background

Hepatocellular Carcinoma (HCC) is currently one of the most lethal malignancies worldwide. Of note, the five-year overall survival (OS) rate of HCC patients remains as low as 5% (Yang and Roberts, 2010; Yang et al., 2019). Therefore, there was an urgent need to identify novel and early biomarkers for HCC. With the development of RNA-sequencing methods, more than 95% of the human genome had been validated to be non-coding genes, including snoRNAs, microRNAs (miRNAs or miRs), piRNAs, and long non-coding RNAs (lncRNAs). Among these ncRNAs, lncRNAs had been demonstrated to be dysregulated and associated with the progression of HCC. For example, lncRNA metabolism-induced tumor activator 1 (MITA1) promoted HCC metastasis. LINC01138 promotes HCC via activating PRMT5 (Li et al., 2018). Exploring the clinical significance and molecular functions of lncRNAs could provide new clues for the identification of novel biomarkers for HCC.

Pseudogenes, a special class of lncRNAs, were originally derived from functional genes. Recent studies had demonstrated that pseudogenes were linked to the progression of human cancers through regulating the expression of oncogenes or tumor suppressors in tumor cells. For example, ANXA2P2 was overexpressed in HCC and promoted HCC migration and invasion (Liu et al., 2019). Pseudogene OCT4-PG1 regulated the multidrug resistance of chronic myeloid leukemia though OCT-4 protein expression (Lettnin et al., 2019). Silencing of pseudogene NANOGP8 suppressed gastric cancer growth via transactivating DBC1 (Li et al., 2019). Moreover, emerging studies demonstrated that the dysregulation of pseudogenes could predict the prognosis of human cancers. For instance, higher expression of pseudogene PTTG3P indicated poor prognosis in breast cancer (Lou et al., 2019). However, the functions and molecular mechanisms of pseudogenes in HCC require further investigation.

DUXAP8 is a novel lncRNAs and has been revealed to be upregulated in multiple human cancers, including HCC, bladder cancer (Lin et al., 2018), and esophageal squamous cell cancer (Xu et al., 2018). However, its roles in HCC have remained unclear. The current study aimed to investigate the expression levels of DUXAP8 between HCC and normal samples and the correlation between its expression and the prognosis of HCC patients using public datasets. Furthermore, we conducted loss-of-function assays to detect the effect of DUXAP8 on HCC proliferation, migration, and invasion. The findings of this study suggest that DUXAP8 could be a novel and suitable predictive biomarker for the risk assessment of recurrence or metastasis of HCC patients but may not be effective in predicting the efficacy of targeted drugs.

## Materials and Methods

### KM Plotter Database

The Kaplan–Meier (KM) plotter^1^ database assesses the effects of 54,675 genes on survival in 18,674 cancer types. The present study defined DUXAP8 high and low groups using the cutoff that was determined by the KM plotter database. Also, the information on clinicopathological variables, including Mutation burden, Grade, and Stage, were also extracted from this database. The number of cases, hazard ratios (HRs), 95% confidence intervals (CIs), and log-rank *p*-values were extracted from the KM plotter webpage.

### qRT-PCR Assays

TRIZOL reagent was used to extract total RNA. A NanoDrop2000c was used to determine RNA quantity. A Reverse Transcription Kit (Takara, China) was used to reverse-transcribe RNA to cDNA. The ABI 7500 was used to conduct the quantificational real-time polymerase chain reaction (qRT-PCR) assays. We normalized the expression of target gene against GAPDH and U6. The primers for qRT-PCR were DUXAP8-F 5′-AGGATGGAGTCTCGCTGTATTGC-3′, DUXAP8-R 5′-GGAGGTTTGTTTTCTTCTTTTTT-3′; GAP DH-F 5′-GGGAGCCAAAAGGGTCAT-3′, GAPDH-R 5′-GA GTCCTTCCACGATACCAA-3′; miR-490-5p-F 5′-CATGGAT CTCCAGGTGG-3′, miR-490-5p-R 5′-TGGTGTCGTGGAGT CG-3′; U6-F 5′-CTCGCTTCGGCAGCACA-3′, U6-R 5′-AAC GCTTCACGAATTTGCGT-3′.

### Cells

We obtained HCC cells (Huh-7, HepG2) from the Institute of Biochemistry and Cell Biology of the Chinese Academy of Sciences. We cultured the cells in RPMI 1640 or DMEM supplemented with 100 U/mL penicillin, 10% fetal bovine serum, and 100 mg/mL streptomycin at 37°C with 5% CO_2_.

### Transfections

We seeded HCC cells in six-well plates with 5 × 10^5^ cells per well. We then used 100 nmol/L siRNA to transfect the cells the next day. The transfections were conducted with 2000 Lipofectamine. After 48 h, the cells were collected for the subsequent experiments. The siRNA sequence for siDUXAP8 was GGAACTTCCCAAACCTCCATGATTT and, for siBUB1, CGAAGAGUGAUCACGAUUU.

### Cell Viability

We seeded Huh-7 and HepG2 cells in 96-well plates with 3000 cells per well. We then used specific or control siRNA to transfect the cell. Cell Proliferation Reagent Kit I was used to assess the cell viability. We conducted each experiment independently three times.

### Flow Cytometry

We transfected HepG2 cells using specific or control siRNA. We then used the Cycle TESTTM PLUS DNA Reagent Kit to stain the cells. For each phase of G0-G1, S, and G2-M phase, we calculated the cell percentages according to the relative DNA content with a FACScalibur flow cytometer (BD, CA, United States) and analyzed with ModFit software (Verity Software House, ME, United States) according to the manufacturer’s procedure. To analyze apoptosis, we collected HepG2 cells 48 h after transfection and stained them with FITC, annexin V, and propidium iodide. FACScan was then used to analyze the cells. We counted the number of the four types of cells, i.e., viable cells, dead cells, early apoptotic cells, and late apoptotic cells.

### Western Blot Assays

Ten percent SDSPAGE was used to separate cell protein lysates. We transferred them to NC membranes of 0.22 μm and incubated them with specific antibodies. Quantification was performed by densitometry using an enhanced chemiluminescence (ECL) chromogenic substrate. We detected the target proteins using antibodies against BUB1 (ab9000, Abcam, Cambridge, United Kingdom). Densitometry was also used to quantify protein bands. We used GAPDH as loading control.

### Statistical Analysis

SPSS software 22.0 was used to perform the statistical analyses. The numerical data were presented as mean ± standard deviation (SD) of at least three determinations. A paired-samples *t*-test was performed to test the difference in DUXAP8 expression between adjacent normal tissues and PC cancer tissues. The Chi-square test was used to evaluate the relationship between clinicopathologic characteristics and DUXAP8. Kaplan–Meier analysis was used to analyze survival data, and then a log-rank test was performed on the results. Two-way ANOVA was used for multiple group comparison, and a linear regression test and Pearson test were used for correlation analyses. In this paper, a statistically significant difference was demonstrated by *p* values ≤0.05.

## Results

### DUXAP8 Is Upregulated in HCC Tissues

**FIGURE 1:**
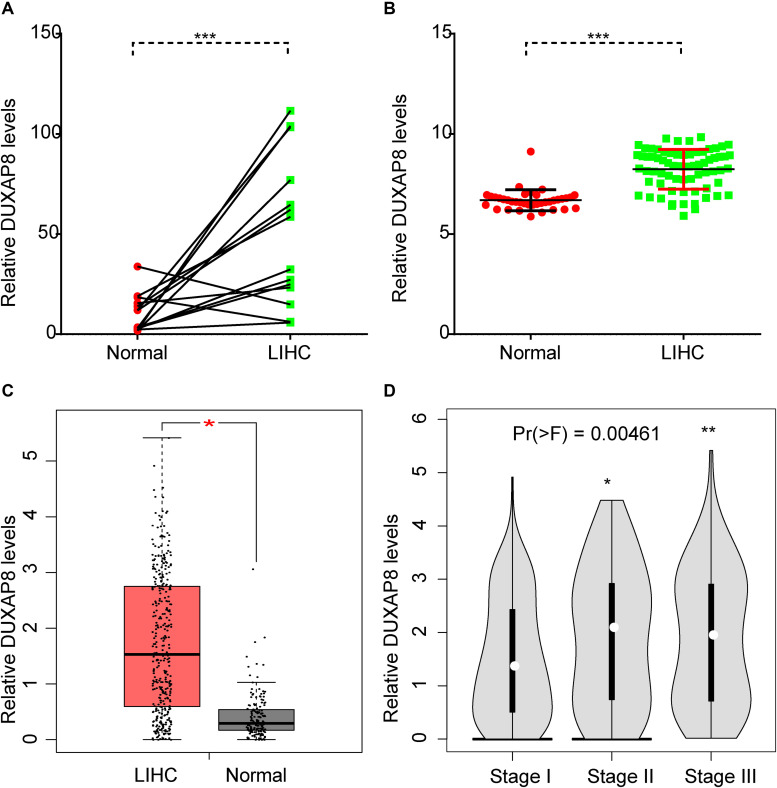
DUXAP8 was upregulated in HCC. **(A,B)** DUXAP8 expression in HCC tissues was found to be higher than in normal tissue by analyzing GSE84402 and GSE121248. **(C)** DUXAP8 expression in HCC tissues was found to be higher than in normal tissue by analyzing TCGA. **(D)** DUXAP8 expression levels were upregulated in Stage II/III compared to Stage I samples. Significance was defined as *p* < 0.05 (*p < 0.05; **p < 0.01; ***p < 0.001).

In order to understand the prognostic value of DUXAP8 in HCC, we performed an integrative analysis of HCC microarray profiles, including GSE84402 and GSE121248 datasets. As shown in Figures 1A,B, the results showed that DUXAP8 was overexpressed in HCC samples compared to normal tissues using GSE84402 (*p* < 0.001) and GSE121248 datasets (*p* < 0.001). Subsequently, the TCGA dataset was also used to determine the expression pattern of DUXAP8 in HCC. We also found upregulated DUXAP8 expression in HCC samples versus adjacent normal tissues (Figure 1C, *p* < 0.05). Very interestingly, we observed that DUXAP8 was upregulated in Stage II/III HCC samples compared to Stage I HCC samples (Figure 1D, *p* < 0.0 and *p* < 0.005). These results suggested that lncRNA DUXAP8 may play a regulatory role in HCC tumorigenesis and development.

### Upregulation of DUXAP8 Expression Is Associated With Poor Prognosis of HCC

**FIGURE 2:**
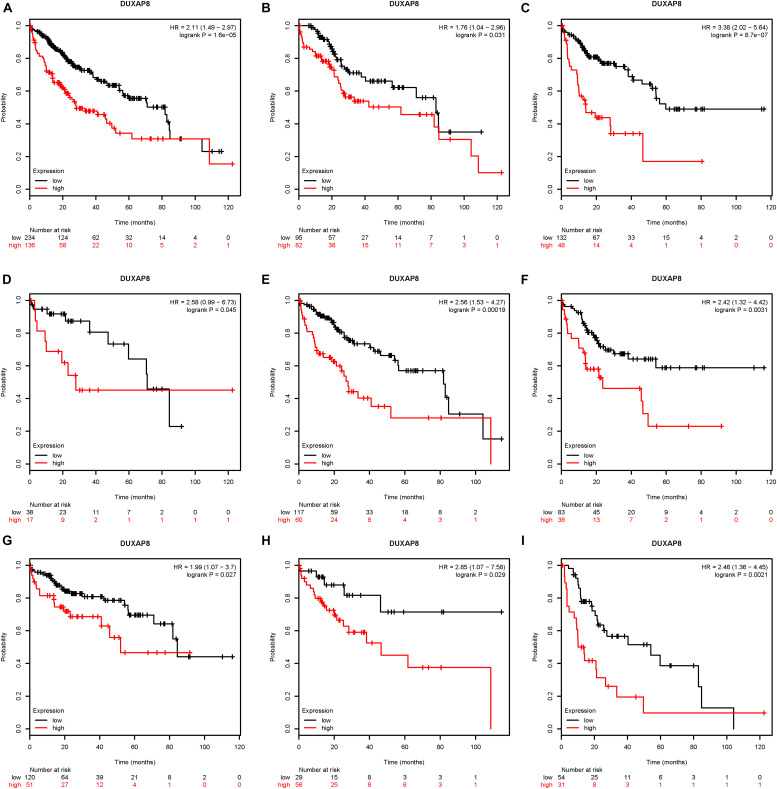
Upregulation of DUXAP8 expression is associated with poor prognosis of HCC. **(A)** Upregulation of DUXAP8 expression is associated with shorter OS of HCC. **(B–I)** increased DUXAP8 expression predicted poor OS in patients with low mutation burden **(B)**, high mutation burden **(C)**, Grade I **(D)**, Grade II **(E)**, Grade III **(F)**, Stage I **(G)**, Stage II **(H)**, and Stage III **(I)** HCC.

Next, the association between DUXAP8 expression and OS time and relapse-free survival (RFS) time were calculated by using the KM survival Plotter database. Kaplan-Meier survival curve analysis indicated that higher expression of DUXAP8 was significantly correlated with shorter OS time in patients with HCC (Figure 2A).

Furthermore, we analyzed the association between DUXAP8 expression and OS time or RFS time in HCC patients according to clinicopathological variables, including Mutation burden, Grade, and Stage. The results indicated that increased DUXAP8 expression predicted poor OS in patients with low Mutation burden (Figure 2B), high Mutation burden (Figure 2C), Grade I (Figure 2D), Grade II (Figure 2E), Grade III (Figure 2F), Stage I (Figure 2G), Stage II (Figure 2H), and Stage III (Figure 2I) HCC. Taken together, these results showed that DUXAP8 could be a potential biomarker for HCC.

### Co-expressed Genes of DUXAP8 and Pathway Analyses

**FIGURE 3:**
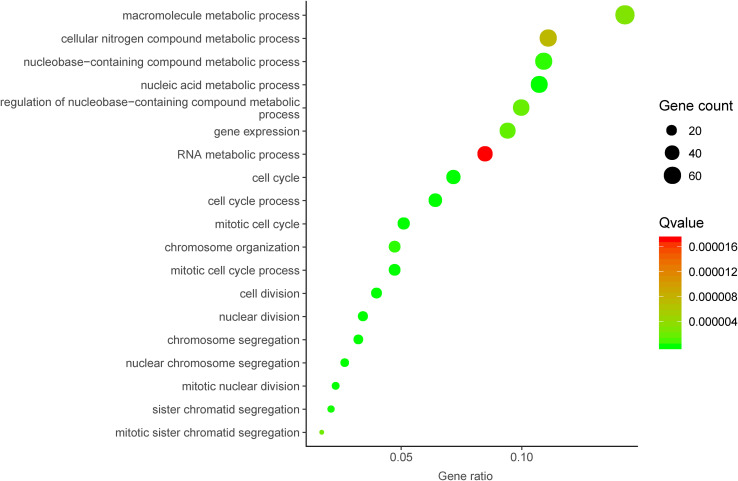
Bioinformatics analysis of DUXAP8 in HCC.

To further investigate the underlying molecular mechanism of DUXAP8 in HCC, we first predicted the co-expressed genes of DUXAP8 using the cBioPortal for Cancer Genomics^2^. The top 200 most significant co-expressed genes were extracted from the cBioPortal and MEM database for further analysis. The top 200 most significant co-expressing genes of lncRNA DUXAP8 were then used to predict the potential functions of DUXAP8 using the DAVID database^3^. GO analysis showed that DUXAP8 was significantly associated with the regulation of multiple cell-cycle-related biological processes, including the cell cycle, the mitotic cell cycle, the mitotic cell cycle process, nuclear division, chromosome segregation, cell division, the nucleic acid metabolic process, nuclear chromosome segregation, mitotic nuclear division, sister chromatid segregation, chromosome organization, the nucleobase-containing compound metabolic process, gene expression, the regulation of nucleobase-containing compound metabolic process, mitotic sister chromatid segregation, the macromolecule metabolic process, the cellular nitrogen compound metabolic process, and the RNA metabolic process (Figure 3).

### Construction of the DUXAP8-Mediated Protein–Protein Interaction Network in HCC

**FIGURE 4:**
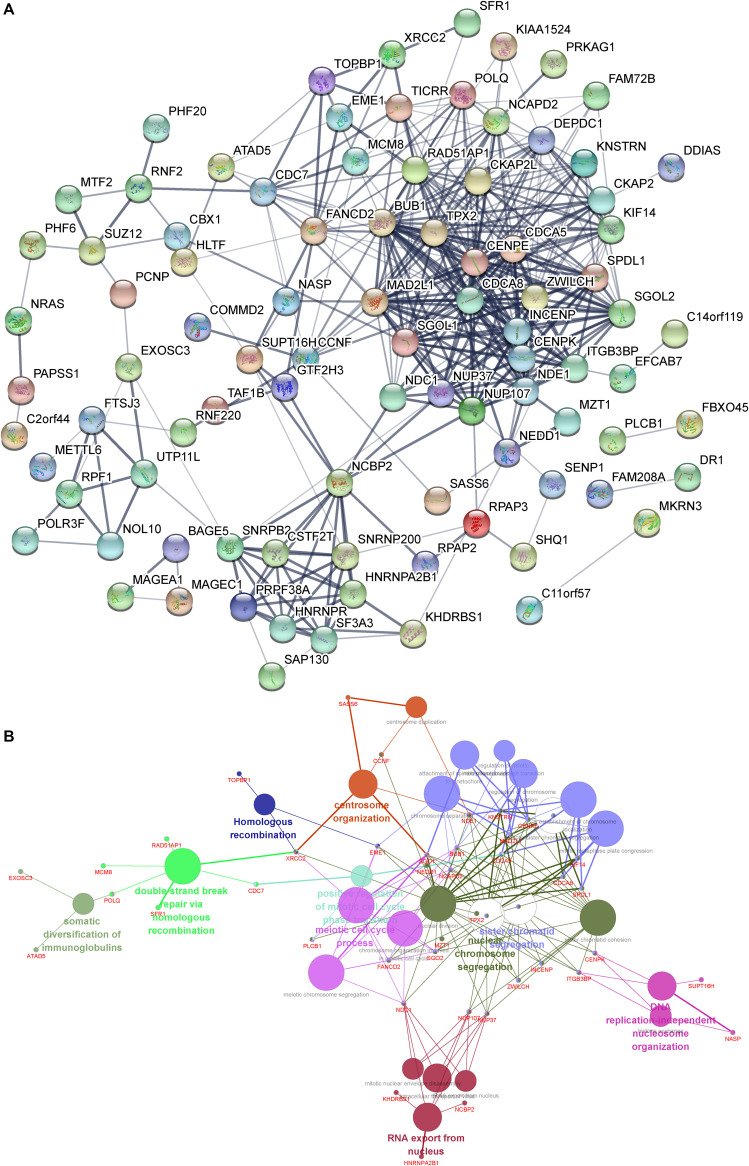
**(A)** Construction of the DUXAP8-mediated PPI network in HCC. **(B)** GO and KEGG pathway analysis of the DUXAP8-mediated hub PPI network in HCC.

In order to understand the interactions among DUXAP8 co-expressing genes, we also constructed a DUXAP8-mediated Protein–Protein Interaction (PPI) network in HCC using the STRING database (combined score >0.4). As present in Figure 4A, this network contained 35 nodes and 182 edges. Several proteins were identified as key regulators of this network, including CDCA8, CENPK, DEPDC1, INCENP, NUP37, NDE1, BUB1, CDC7, CKAP2, SGOL2, CKAP2L, ITGB3BP, KIF14, NCAPD2, TPX2, and ZWILCH, which connected with more than 10 proteins in this network.

### Gene Ontology and Kyoto Encyclopedia of Genes and Genomes Pathway Analysis of the DUXAP8-Mediated Hub PPI Network in HCC

We next performed gene ontology (GO) and Kyoto Encyclopedia of Genes and Genomes (KEGG) pathway analysis of the DUXAP8-mediated hub PPI network in HCC using Cytoscape’s ClueGo plug-in. Only significant biological processes and pathways (*p* ≤ 0.05) were shown. Our results showed that the DUXAP8-mediated hub PPI network was significantly associated with the regulation of centrosome organization, homologous recombination, the meiotic cell cycle process, sister chromatid segregation, nuclear chromosome segregation, and RNA export from the nucleus. These results were consistent with the aforementioned bioinformatics analysis showing that DUXAP8 was involved in regulating cell cycle processes in HCC (Figure 4B). A series of cell cycle regulators were included in this network, including BUB1, CDCA5, CDCA8, SPDL1, NEDD1, NCAPD2, SGO1, TPX2, CENPE, KNSTRN, NDE1, MAD2L1, and KIF14 (Figure 4B).

### Silencing of DUXAP8 Suppressed Proliferation and Induces Apoptosis in HCC Cells

**FIGURE 5:**
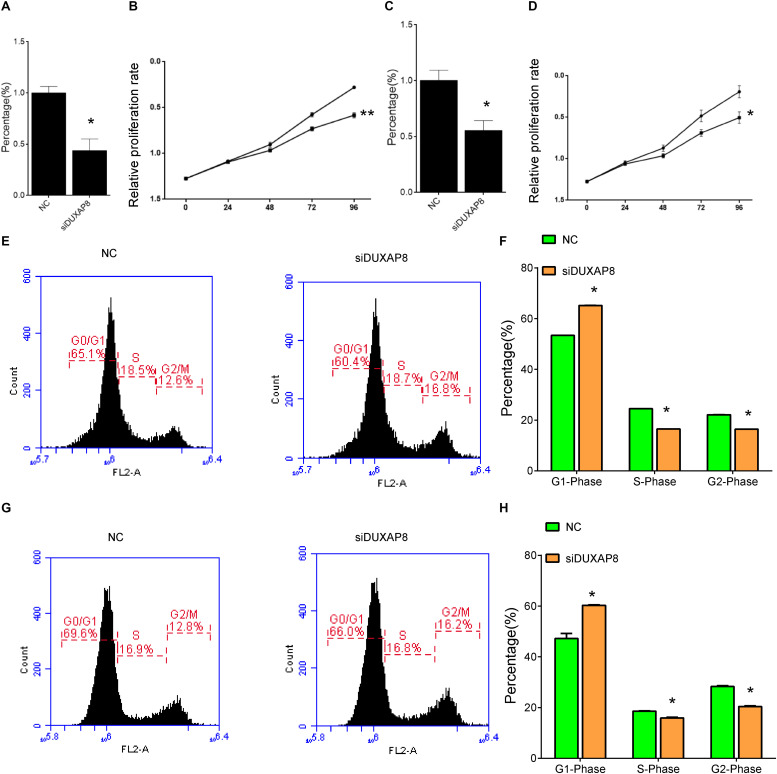
Knockdown of DUXAP8 suppressed HCC Cell Viability. **(A,C)** The endogenous DUXAP8 expressions in Huh-7 **(A)** and HepG2 **(C)** cells transfected with siDUXAP8 were reduced. **(B,D)** The results showed that the silencing of DUXAP8 significantly suppressed cell proliferation in both Huh-7 **(B)** and HepG2 **(D)**. **(E,F)** The results showed that the silencing of DUXAP8 significantly suppressed the cell cycle in Huh-7 cells. **(G,H)** The results showed that the silencing of DUXAP8 significantly suppressed the cell cycle in HepG2 cells. All assays were conducted with at least three determinations. Statistical comparisons between groups of normalized data were performed using Students’ *t*-test. Significance was defined as *p* < 0.05 (*p < 0.05; **p < 0.01; ***p < 0.001).

Bioinformatics analysis showed that DUXAP8 was involved in regulating cancer proliferation in HCC. To further validate the biological function of DUXAP8 in HCC cells, we conducted loss-of-function assays by using specific siRNAs against DUXAP8. As shown in Figure 5, we found that the endogenous DUXAP8 expressions in Huh-7 and HepG2 cells transfected with siDUXAP8 were reduced by 53.4% (Figure 5A, *p* < 0.05) and 47.6% (Figure 5C, *p* < 0.05), respectively. Furthermore, CCK-8 assay was applied to detect the effect of DUXAP8 on cell proliferation in HCC. The results showed that the silencing of DUXAP8 significantly suppressed cell proliferation in both Huh-7 (Figure 5B, *p* < 0.01) and HepG2 (Figure 5D, *p* < 0.05).

**FIGURE 6:**
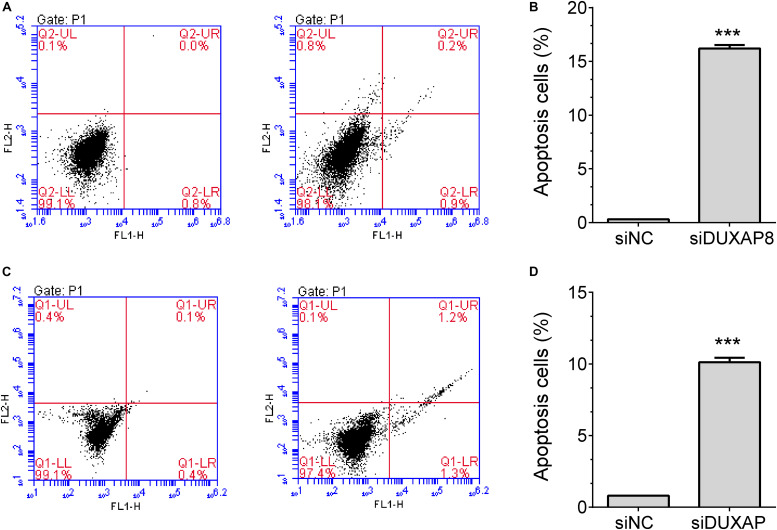
Knockdown of DUXAP8 induced HCC cell apoptosis. **(A,B)** The results showed that the silencing of DUXAP8 significantly induced cell apoptosis in Huh-7 cells. **(C,D)** The results showed that the silencing of DUXAP8 significantly induced cell apoptosis in HepG2 cells. All assays were conducted with at least three determinations. Statistical comparisons between groups of normalized data were performed using Students’ *t*-test. Significance was defined as *p* < 0.05 (*p < 0.05; **p < 0.01; ***p < 0.001).

In addition, flow cytometry was used to detect the influence of DUXAP8 on cell cycle progression and apoptosis in HCC. The results showed that the proportion of the G1 phase was significantly induced and the proportion of S and G2 phase was significantly reduced in both Huh-7 (Figures 5E,F, *p* < 0.05) and HepG2 (Figures 5G,H, *p* < 0.05) cells, suggested that knockdown of DUXAP8 could arrest the cell cycle at the G1/G0 phase. Apoptosis assay showed that apoptosis was significantly induced in Huh-7 and HepG2 transfected with siDUXAP8: by 15.1% (Figures 6A,B, *p* < 0.001) and 9.83% (Figures 6C,D, *p* < 0.001) compared to the negative control group.

### DUXAP8 Regulated Multiple Cell Cycle Regulators in HCC

**FIGURE 7:**
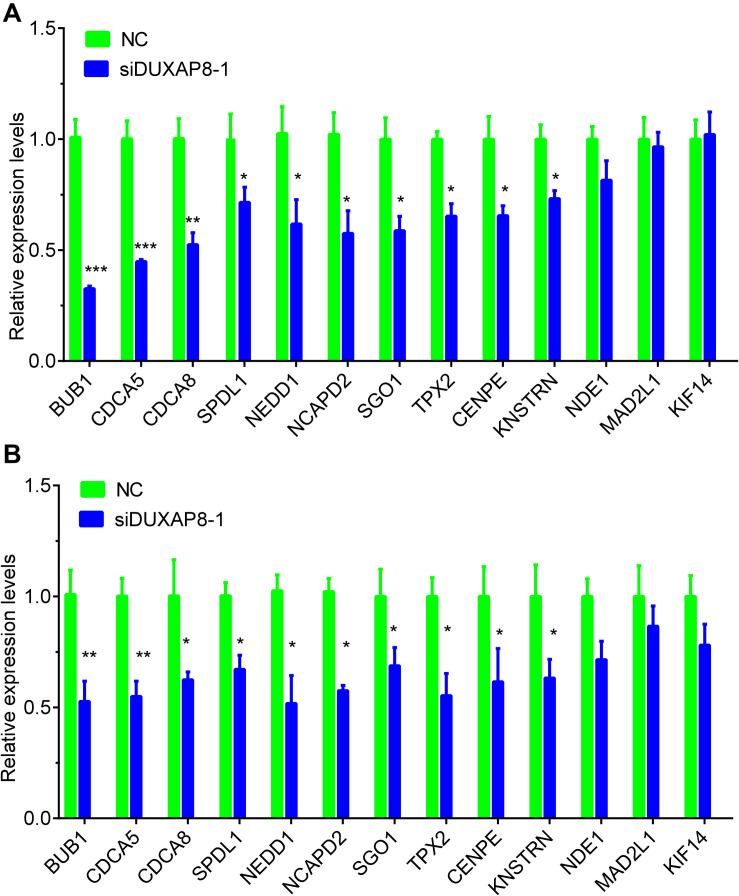
Knockdown of DUXAP8 suppressed Cell cycle regulators. **(A,B)** Knockdown of DUXAP8 suppressed the expression of Cell cycle regulators in both Huh-7 **(A)** and HepG2 **(B)**. All assays were conducted at least three determinations. Statistical comparisons between groups of normalized data were performed using Students’ t-test. Significance was defined as *p* < 0.05 (*p < 0.05; **p < 0.01; ***p < 0.001).

Furthermore, we detected the expression of cell cycle regulators in HCC after knockdown of DUXAP8 expression. The results showed that knockdown of DUXAP8 significantly suppressed the expression levels of BUB1, CDCA5, CDCA8, SPDL1, NEDD1, NCAPD2, SGO1, TPX2, CENPE, KNSTRN, and NDE1. However, knockdown of DUXAP8 did not affect the expressions of MAD2L1 and KIF14 compared to the control group in both Huh-7 and HepG2 cells (Figures 7A,B).

### DUXAP8 Promoted BUB1 Expression Through Sponging MiR-490-5p

Among these genes, BUB1 was reported as a key regulator in multiple cancer progression, including HCC, and was selected as the downstream target of DUXAP8. Starbase was then used to predict the potential miRNAs mediating the connection between BUB1 and DUXAP8, and MiR-490-5p was identified as the candidate miRNA involved in regulating both BUB1 and DUXAP8.

**FIGURE 8:**
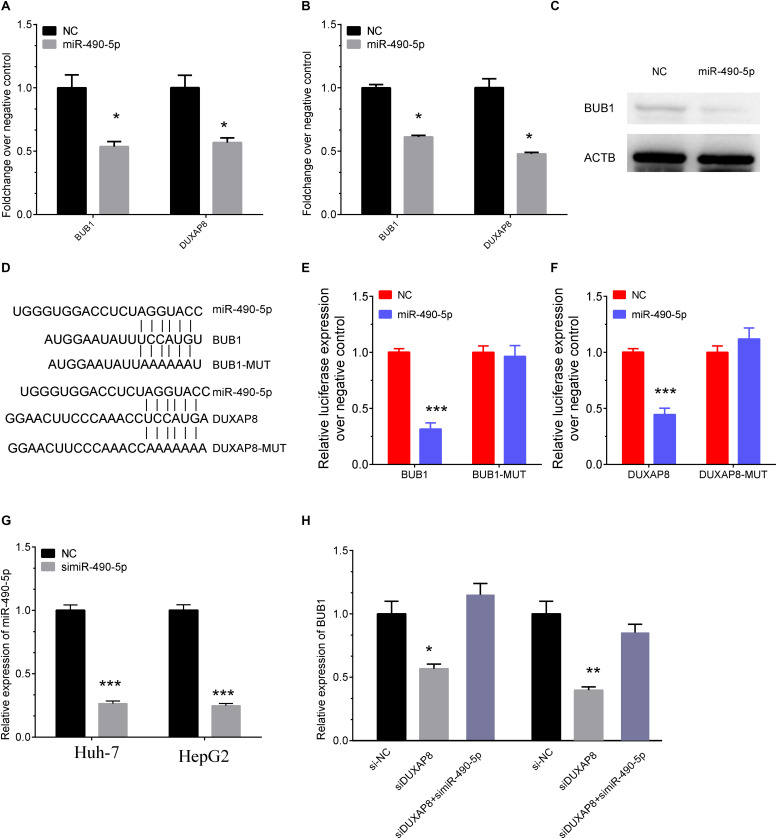
DUXAP8 promoted BUB1 expression through sponging MiR-490-5p. **(A,B)** The RNA levels of BUB1 and DUXAP8 were significantly reduced after overexpressing miR-490-5p in Huh-7 **(A)** and HepG2 **(B)** cells by using RT-PCR assay. **(C)** Western blot assay also showed that the protein levels of BUB1 were significantly decreased in Huh-7 cells transfected with miR-490-5p compared to the control group. **(D)** Bioinformatics analysis predicted the direct interaction between miR-490-5p and BUB1 or DUXAP8. **(E,F)** Luciferase reporter assay revealed that luciferase activity was significantly repressed in the constructs of the DUXAP8 **(E)**- and BUB1-3′UTR **(F)** when co-transfected with the corresponding miRNAs compared with NC, whereas the mutated 3′UTR did not show a significant response to miR-490-5p. **(G)** The knockown efficiency of MiR-490-5p in HCC cells. **(H)** The expression levels of BUB1 after knockdown of DUXAP8 or miR-490-5p. All assays were conducted at with least three determinations. Statistical comparisons between groups of normalized data were performed using Students’ *t*-test. Significance was defined as *p* < 0.05 (*p < 0.05; **p < 0.01; ***p < 0.001).

We then detected the expression levels of BUB1 and DUXAP8 after overexpressing miR-490-5p in Huh-7 and HepG2 cells. As presented in Figure 8, our results showed the RNA levels of BUB1 and DUXAP8 were significantly reduced after overexpressing miR-490-5p in Huh-7 (Figure 8A, *p* < 0.05) and HepG2 (Figure 8B, *p* < 0.05) cells according to real-time polymerase chain reaction (RT-PCR) assay. Western blot assay also showed that the protein levels of BUB1 were significantly decreased in Huh-7 cells transfecting with miR-490-5p compared to the control group (Figure 8C).

Furthermore, direct interaction between miR-490-5p and BUB1 or DUXAP8 was detected using luciferase assays (Figure 8D). The luciferase reporter assay revealed that luciferase activity was significantly repressed in the constructs of DUXAP8 (Figure 8E, *p* < 0.001)- and BUB1-3’UTR (Figure 8F, *p* < 0.001) when co-transfected with the corresponding miRNAs compared with NC, whereas the mutated 3’UTR did not show a significant response to miR-490-5p. Moreover, the results showed that BUB1 was suppressed after DUXAP8 knockdown and that the DUXAP8 knockdown-mediated suppression of BUB1 was reversed by miR-490-5 inhibitors in HCC cells (Figures 8G,H). Thus, these results showed that DUXAP8 sponges miR-490-5p to enhance BUB1 expression.

### DUXAP8 Promoted Cell Proliferation Through BUB1 in HCC

**FIGURE 9:**
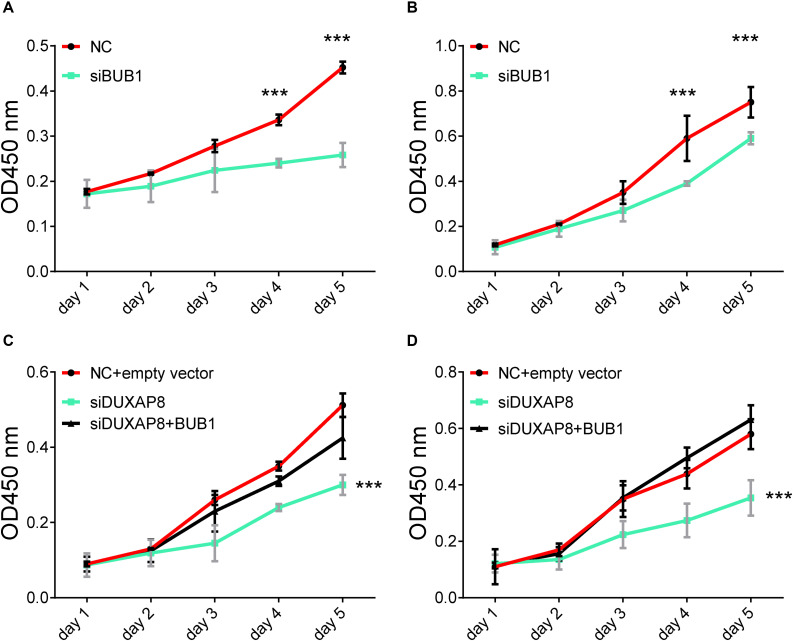
Overexpresion of BUB1 reverses the effects of DUXAP8 knockdown in HCC. **(A,B)** Knockdown of BUB1 decreased cell proliferation in Huh-7 and HepG2. **(C,D)** Overexpression of BUB1 reversed the effects of decreased cell proliferation in DUXAP8 knockdown Huh-7 and HepG2 cells. All assays were conducted with at least three determinations. Statistical comparisons between groups of normalized data were performed using Students’ *t*-test. Significance was defined as *p* < 0.05 (*p < 0.05; **p < 0.01; ***p < 0.001).

In order to further validate the biological functions of BUB1 in HCC, we conducted gain of function studies by transient transfection with BUB1 mimics in Huh-7 and HepG2 cells. As presented in Figure 9, we found that knockdown of BUB1 significantly inhibited the proliferation of Huh-7 and HepG2 cells: by 48% (Figure 9A, *p* < 0.001) and 43% (Figure 9B, *p* < 0.001) compared with the NC group, respectively.

To test whether DUXAP8 promoted cell migration and invasion of PCa through BUB1, we conducted recuse experiments. Huh-7 (Figure 9C, *p* < 0.001) and HepG2 (Figure 9D, *p* < 0.001) cells co-transfected with siDUXAP8 and BUB1 attenuated cell proliferation by 65%, which led to an approximately 12% increase in the cell proliferation rate compared to the cells only transfected with siDUXAP8. Taken together, these results suggested that DUXAP8 promoted cell proliferation through BUB1 in HCC.

## Discussion

Emerging studies had demonstrated that lncRNAs, such as pseudogenes, played a crucial role in regulating cancer progression. In HCC, lncRNAs were involved in regulating cancer growth, chemoresistance, and metastasis. For example, lncRNA PSTAR suppressed HCC tumorigenesis through activating p53 signaling by inhibiting hnRNP K deSUMOylation (Qin et al., 2020). Pseudogene DUXAP8 is a novel lncRNA that has been reported to be overexpressed in a series of human cancers, including glioma and renal cell carcinoma (Huang et al., 2018). However, the roles of DUXAP8 in HCC have remained unclear, and whether DUXAP8 could be a novel biomarker for HCC remained to be further investigated. The present study, for the first time, showed that DUXAP8 was significantly overexpressed in HCC samples. Higher expression of DUXAP8 was remarkably associated with worse prognosis in patients with HCC. Furthermore, loss-of-function assays showed that knockdown of DUXAP8 significantly suppressed HCC proliferation and cell cycle but induced cell apoptosis. These results suggested that DUXAP8 functioned as an oncogene in HCC.

The mechanisms of DUXAP8 regulation of tumor progression have been shown to differ in different types of human cancers. DUXAP8 was initially reported to promote non-small-cell lung cancer proliferation and invasion by epigenetically silencing EGR1 and RHOB (Sun et al., 2017). In gastric cancer, DUXAP8 could promote tumor growth by interacting with the PRC2 complex to inhibit PLEKHO1 expression (Ma et al., 2017). In bladder cancer, knockdown of DUXAP8 inhibited tumor proliferation through PTEN (Lin et al., 2018). In pancreatic cancer, DUXAP8 enhanced cancer cell proliferation by epigenetically silencing CDKN1A and KLF2 (Lian et al., 2018). The present study demonstrated the oncogenetic roles of DUXAP8 in HCC. In order to investigate the mechanisms of DUXAP8 in HCC, we conducted co-expression and bioinformatics analysis of DUXAP8, and the results showed that DUXAP8 was involved in regulating multiple cell cycle-related biological processes, such as the cell cycle, the mitotic cell cycle, nuclear division, chromosome segregation, and cell division. By constructing a PPI network, we found that DUXAP8 could regulate multiple cell cycle regulators in HCC, including BUB1, CDCA5, CDCA8, SPDL1, NEDD1, NCAPD2, SGO1, TPX2, CENPE, KNSTRN, and NDE1. RT-PCR validation showed that knockdown of DUXAP8 significantly suppressed the expression of these genes in HCC. Furthermore, the present study showed that DUXAP8 could promote BUB1 expression through sponging miR-490-5p.

BUB1 is a cell cycle regulator and is involved in regulating chromosome congression by phosphorylating the mitotic checkpoint complex and activating the spindle checkpoint. BUB1 has been found to be upregulated and to promote cancer proliferation and metastasis in various types of cancers, including pancreatic ductal adenocarcinoma (Piao et al., 2019) and ovarian cancer. MiR-490-5p has been recognized as a tumor suppressor in human cancers, including HCC (Weidle et al., 2020). For example, Fang et al. found that MiR-490-5p suppressed HCC metastasis by downregulating E2F2 and ECT2 (Fang et al., 2018). Moreover, ROBO1 and SOX2 were also identified as the direct targets of MiR-490-5p in HCC (Chen et al., 2019). Of note, a recent study showed that MiR-490-5p could inhibit HCC proliferation and invasion by targeting BUB1 (Xu et al., 2017). The present study, for the first time, demonstrated that MiR-490-5p could directly bind to lncRNA DUXAP8. DUXAP8 serves as a sponge of MiR-490-5p to promote the expression of BUB1. Moreover, this study confirmed that DUXAP8 regulated HCC proliferation through BUB1.

## Conclusion

In summary, we report that lncRNA DUXAP8 was upregulated in tumor samples and served as an oncogene in HCC. The knockdown of DUXAP8 significantly suppressed cell proliferation and the cell cycle but induced cell apoptosis in HCC. Mechanically, the present study showed that DUXAP8 serves as a sponge of MiR-490-5p to promote the expression of BUB1 in HCC. Although the underlying regulatory mechanisms of DUXAP8 in HCC require further investigation, this study, for the first time, showed that DUXAP8 can serve as a new therapeutic target for HCC.

## Data Availability Statement

The raw data supporting the conclusions of this article will be made available by the authors, without undue reservation, to any qualified researcher.

## Author Contributions

RT: conception, design, and administrative support. HZ and KC: provision of study materials or patients, data analysis, and interpretation. KC, CZ, and LR: collection and assembly of data. All authors wrote and approved the final version of the manuscript.

## Conflict of Interest

The authors declare that the research was conducted in the absence of any commercial or financial relationships that could be construed as a potential conflict of interest.

## Footnotes

1 http://kmplot.com/analysis/

2 http://www.cbioportal.org/

3 https://david.ncifcrf.gov/tools.jsp
